# Bioactive Amines in Wines. The Assessment of Quality Descriptors by Flow Injection Analysis with Tandem Mass Spectrometry

**DOI:** 10.3390/molecules27248690

**Published:** 2022-12-08

**Authors:** Aina Mir-Cerdà, Javier Saurina, Sònia Sentellas

**Affiliations:** 1 Department of Chemical Engineering and Analytical Chemistry, Universitat de Barcelona, Martí i Franquès 1-11, E08028 Barcelona, Spain; 2 Research Institute in Food Nutrition and Food Safety, Universitat de Barcelona, Recinte Torribera, Av. Prat de la Riba 171, Edifici de Recerca (Gaudí), Santa Coloma de Gramenet, E08921 Barcelona, Spain; 3 Serra Húnter Fellow Programme, Generalitat de Catalunya, Via Laietana 2, E08003 Barcelona, Spain

**Keywords:** biogenic amines, bioactivity, adverse effects, health, food quality, FIA-MS/MS, wines

## Abstract

Biogenic amines (BAs) occur in a wide variety of foodstuffs, mainly from the decomposition of proteins by the action of microorganisms. They are involved in several cellular functions but may become toxic when ingested in high amounts through the diet. In the case of oenological products, BAs are already present in low concentrations in must, and their levels rise dramatically during the fermentation processes. This paper proposes a rapid method for the determination of BAs in wines and related samples based on precolumn derivatization with dansyl chloride and further detection by flow injection analysis with tandem mass spectrometry. Some remarkable analytes such as putrescine, ethanolamine, histamine, and tyramine have been quantified in the samples. Concentrations obtained have shown interesting patterns, pointing out the role of BAs as quality descriptors. Furthermore, it has been found that the BA content also depends on the vinification practices, with malolactic fermentation being a significant step in the formation of BAs. From the point of view of health, concentrations found in the samples are, in general, below 10 mg L^−1^, so the consumption of these products does not represent any special concern. In conclusion, the proposed method results in a suitable approach for a fast screening of this family of bioactive compounds in wines to evaluate quality and health issues.

## 1. Introduction

Biogenic amines (BAs) are low molecular mass organic compounds that participate in the normal metabolic pathways of living beings. According to the number of amino groups, BAs are classified into monoamines (such as histamine, tyramine, and ethanolamine), diamines (putrescine and cadaverine), and polyamines (spermine and spermidine). Structurally, the hydrocarbon skeleton can be aliphatic, aromatic, and heterocyclic (see structures of the studied amines in the Supplementary Materials Section, Figure S1). The formation of BAs is often associated with the degradation of protein and amino acid precursors in metabolic processes by decarboxylases coming from several types of microorganisms [1]. Hence, protein-rich foodstuffs, including fish, meat, vegetables, and fermented products—such as dairy products, beer, and wine—may contain low concentrations of BAs as typical components [2,3,4,5,6]. Anyway, high BA levels are indicators of food spoilage by putrefaction processes or products obtained/manufactured under poor sanitary conditions.

In wines, the most abundant amines are histamine, putrescine, tyramine, and ethanolamine [7,8,9]. They are often in concentrations ranging from 1 to 10 mg L^−1^, although higher values are found in some cases. Other amines may be present at sub-mg L^−1^ levels, such as cadaverine, phenylethylamine, tryptamine, agmatine, spermine, and spermidine.

Histamine was first identified in wines in the 1950s. In the following years, other amines were detected, such as tyramine, putrescine, and cadaverine. Nowadays, the toxicological implications of BAs have been thoroughly studied concluding that rich-amine products may be harmful, especially for sensitive individuals, when their biological activities become toxic if ingested in high amounts [10,11]. For instance, histamine, which is one of the most remarkable amines in wines, is responsible for psychoactive (headache, palpitations, and itching), vasoactive (hypotension), cutaneous (rash), and gastrointestinal (nausea, vomiting, and diarrhea) effects. Episodes of histamine (scombroid) poisoning may occur in sensitive individuals after ingesting some mg of this amine [12,13,14]. The histamine toxicity is enhanced in combination with alcohol, other BAs, and some antidepressant and antihypertensive drugs. Tyramine is another important monoamine that induces the release of catecholamine neurotransmitters. Hence, the ingestion of tyramine may increase blood pressure and cardiac frequency. Tyramine has been also considered a trigger of migraine in sensitive individuals. Phenylethylamine (PEA) is present, in general, at sub-mg L^−1^ levels in wine samples, acting as a neuromodulator of catecholaminergic transmission. Interestingly, the resemblance of PEA with amphetamine seems to activate the release of dopamine and endorphins, thus being recognized as a natural antidepressant product [9]. Putrescine is the most abundant diamine in wines, with concentrations often ranging from 2 to 20 mg L^−1^. Like cadaverine, they do not represent any health concerns but high concentrations negatively modify the flavor, providing unpleasant organoleptic attributes such as rancid, meaty, and vinegary aromas [15]. Natural polyamines such as spermine and spermidine are involved in growth and cell proliferation. Beyond the mentioned physiological functions, high levels may induce uncontrolled cell growth and cytotoxicity [16].

BA levels in wines result in a multifactorial issue, depending on features such as grape varieties, agricultural practices, and vinification [7,17,18], with malolactic fermentation and aging being the most important steps contributing to their formation. In addition, high levels of BAs may evidence hygienic deficiencies during the winemaking process. For instance, red wines are commonly richer than white wines (50% higher or more), probably due to the differences in fermentation and aging conditions [19]. Alcoholic fermentation generates small amounts of certain compounds, such as tyramine. In any case, malolactic fermentation is, by far, the most important step contributing to the formation of amines during winemaking, with bacteria of the genus Oenococcus, Lactobacillus, and Pediococcus being responsible for these processes [20,21,22]. For this reason, new winemaking technologies providing low amine levels based on the choice of suitable microorganism starters, together with the careful control of the fermentation and aging processes are fundamental for preserving the quality of wines [23,24]. Even so, although the concentration of biogenic amines is in a safe range, their determination may result in a reliable strategy for assessing wine quality, so for this purpose, efficient analytical methods are required. Currently, liquid chromatography with ultraviolet–visible (UV/Vis), fluorescence (FLD), or mass spectrometry (MS) detection is the most common technique for the determination of BAs in foodstuffs, including wines [25,26,27]. However, chromatographic methods often involve long analysis times, usually more than 20 min per sample. Alternatively, flow injection analysis (FIA) offers the possibility of drastically reducing the analysis time without the physical separation of analytes, however, a selective detection mode is required to differentiate the compounds under study, among them, and from the other matrix components. Although FIA-MS has been reported to provide good performances for other families of components [28], to our knowledge this strategy has not been previously applied to the determination of BAs in beverages. Hence, the development of high-throughput methods is welcome, especially for rapid control of toxicological and quality issues in a large set of samples.

The quality of a set of oenological products, including musts, wines, and sparkling wines, has been evaluated here based on the BA composition. Samples have been analyzed by a fast-screening flow injection analysis–mass spectrometry (FIA-MS) method for BA profiling, as a novel approach for high-throughput sample analysis. As expected, the potentially most harmful compounds, such as tyramine and histamine, are found at low concentrations so that they do not present any risk to the consumers. Other molecules, such as putrescine, which might affect the organoleptic characteristics of wines, are also found at fairly low concentrations. Beyond the toxicological implications, BA levels seem to depend on the quality of grapes and the neatness of the winemaking processes, which includes the phytochemical treatments used, the productivity per hectare, the state of fruit maturation, the harvesting and transport procedures, and the pressing yield. Generally speaking, traditional procedures in which grapes are cultivated with less extensive practices and without mechanical handling lead to products of better quality since they preserve the product characteristics and minimize chemical and microbiological degradations responsible for the formation of BAs.

## 2. Results

The FIA-MS method has been used to determine the BA concentrations in a set of samples under study. Some analytical parameters have been established to evaluate the performance of the method. The linear range is, in general, at least 10 mg L^−1^ (up to 25 mg L^−1^ for histamine and tyramine), the limits of detection are between 0.1 and 0.8 mg L^−1^, and the repeatabilities are better than 10% for all of the analytes. These values are satisfactory considering the high-throughput screening nature of our proposal. A more detailed table is given in the Supplementary Materials (Table S1).

As detailed below in the experimental section, the samples were analyzed in triplicate, and a table summarizing the average values and the corresponding standard deviations of each determination can be found in the Supplementary Materials (see Table S2). Ethanolamine, putrescine, and tyramine were the most abundant amines in this set of samples, with overall concentrations of ca. 5 mg L^−1^. Average levels of histamine and octopamine were 2.6 and 1.2 mg L^−1^, respectively, while the rest of the amines occurred at sub-mg L^−1^ levels.

**Figure 1:**
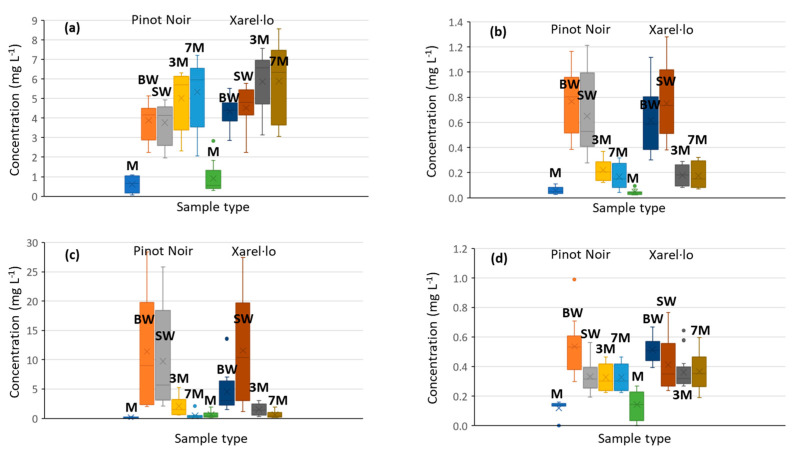
Boxplots with the evolution of the content of biogenic amines according to the sample type for the Pinot Noir and Xarel·lo varieties; (**a**) ethanolamine; (**b**) phenylethylamine; (**c**) putrescine; and (**d**) tryptamine. Sample assignation: musts (M), base wines (BW), stabilized wines (SW), sparkling wines (3 months aged, 3M), sparkling wines (7 months aged, 7M). Independent replicates of each sample have been considered.

Regarding the different types of products, comprising must, base wine, stabilized wine, and sparkling wines subjected to a second fermentation (and aging periods of 3 and 7 months), remarkable differences were encountered in the BA amounts present in must vs. the fermented samples. In general, depending on the amines, concentrations in musts were 2- to 10-fold lower than those found after fermentation, as can be seen in various representative examples depicted in Figure 1, where boxplots with whiskers for each product type for Pinot Noir and Xarel·lo varieties can be seen. Ethanolamine concentrations increased from musts to wines for both Pinot Noir and Xarel·lo, with sparkling wines being the richest products. This pattern was specific to this compound. Phenylethylamine, putrescine, and tryptamine contents increased from musts to wines while diminished during the second fermentation and aging. This behavior can be extended to other amines, such as histamine, tyramine, cadaverine, octopamine, and agmatine.

**Figure 2:**
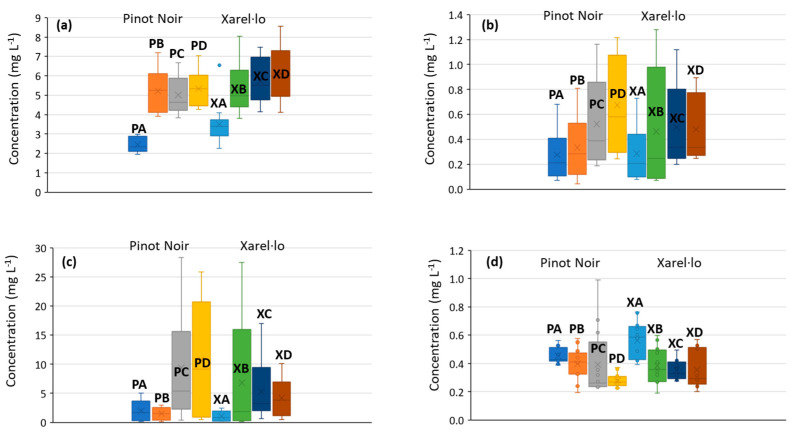
Boxplots depicting the evolution of the content of biogenic amines according to quality for the Pinot Noir and Xarel·lo varieties; (**a**) ethanolamine; (**b**) phenylethylamine; (**c**) putrescine; and (**d**) tryptamine. Sample assignation: A-type Pinot Noir (PA), B-type Pinot Noir (PB), C-type Pinot Noir (PC), D-type Pinot Noir (PD), A-type Xarel·lo (XA), B-type Xarel·lo (XB), C-type Xarel·lo (XC), and D-type Xarel·lo (XD). Independent replicates of each sample have been considered.

As commented in the introduction section, BAs are substantial descriptors of food quality. This is a well-known feature that has already been confirmed here for most of the amines under study (see Figure 2). The top quality (A quality) always displayed the lowest concentrations of ethanolamine, phenylethylamine, putrescine, cadaverine, histamine, octopamine, and tyramine. In general, the second-best quality (B quality) also presented low BA concentrations; in the case of Pinot Noir samples, they were slightly higher than levels found in A-class samples (except for ethanolamine), while Xarel·lo concentrations were closer to those of C and D qualities. Differences among C and D qualities were not so noticeable, although, in general, the poorest class also presented the highest levels.

Finally, as we can observe in the comparative results of Figure 1 and Figure 2, differences in BAs attributable to the grape varieties were less important, at least for the two examples explored in this paper. In all the studies, Pinot Noir and Xarel·lo products followed similar patterns. Some quantitative variations commented on above were assessed statistically using ANOVA. The results demonstrated that differences were statistically significant for ethanolamine, putrescine, octopamine, and tyramine (*p* < 0.05). Conversely, the variety was not influential on the concentration levels of agmatine, tryptamine, phenylethylamine, and cadaverine.

## 3. Discussion

Most of the analytical methods reported in the scientific literature for the determination of BAs in wines and other food matrices rely on liquid chromatography with UV-Vis, FLD, or MS detection. In general, precolumn derivatization with chromogenic and fluorogenic labeling agents, such as 6-aminoquinolyl-N-hydroxysuccinicmidyl carbamate (AQC), o-phthalaldehyde (OPA), fluorenylmethyl chloroformate (FMOC), and dansyl chloride (dansyl-Cl) is applied to enhance sensitivity and facilitate the chromatographic separation under the reversed-phase mode. The resulting methods show excellent analytical parameters in terms of precision, detection limits, and accuracy, as reviewed in various recent publications [25,26,27]. Despite the remarkable performance of chromatographic methods, the analysis time is one of the main limitations, especially for the screening and fast control of a large sample series. In this context, high-throughput methods, such as those based on FIA, are welcome. To date, no FIA method has been published that allows a multicomponent determination of the most significant amines in wines. In this paper, we propose a new method for the quantification of several amines by FIA-MS/MS in which the FIA peak of each compound is monitored from the most sensitive MRM transition. However, the lack of separation could generate matrix effects due to phenomena such as ionic suppression. However, although the quality parameters of the method are somewhat lower than those of the corresponding chromatographic methods [7,29], the final result is satisfactory because very fast and high-quality determinations are achieved.

The role of BAs as the descriptors or biomarkers for different food samples has been emphasized in various interesting reviews. In the previous section, we mentioned some trends and dependencies to be discussed here in more detail. From a toxicological point of view, in very few cases, individual amine concentrations exceeded 15 mg L^−1^ in some of the different stages of wine production (in general, these highest values were obtained for the base and stabilized wines). However, considering that these samples were intended for the production of sparkling wines, in those samples corresponding to the latest steps of the vinification (i.e., the 7-month-aged sparkling wines), BAs hardly exceeded 6 mg L^−1^. As a result, the potential risk of these samples due to the occurrence of some hazardous compounds such as histamine, tyramine, and phenylethylamine is negligible. In parallel, the concentrations in the final products of diamines potentially responsible for some unpleasant taste notes were below 2.0 mg L^−1^ and 0.15 mg L^−1^ for putrescine and cadaverine, respectively. In any case, wine is a complex matrix rich in a wide range of volatile compounds that provide an intense aroma profile. Even though the rancid and rotten notes of putrescine and cadaverine could be recognized at quite low levels from pure standard solutions, they remain undetectable in the wines. In any case, the chemical determination of diamines seems to be an excellent option to control these unpleasant compounds before they affect the sensory attributes of wines. If so, their influence on flavor issues is expected to be irrelevant.

Low BA concentrations were already present in musts before being subjected to transformation processes other than pressing (e.g., ethanolamine from 0.15 to 2.3 mg L^−1^, histamine from 0.06 to 0.3 mg L^−1^, tyramine from 0.3 to 1 mg L^−1^, and putrescine from 0.07 to 1.5 mg L^−1^). Their occurrence was attributed to some degradations in the proteinaceous matrix during grape harvest, transport, and pressing. Factors such as manual harvesting, refrigerated processing, or low pressing yield better preserve product integrity thus minimizing the chemical and microbiological degradations that lead to the formation of BAs. In parallel, malolactic fermentation is one of the most relevant oenological sources of BAs. In this regard, specific bacteria and nutrients can be selected to reduce the formation of biogenic amines at this stage [23,24]. A dramatic BA production after the first alcoholic fermentation was found due to microorganism activity. From this step, the evolution of the content of BAs depended on the nature of each compound. For instance, ethanolamine showed a progressive rise from must to sparkling wine (Figure 1a). This finding was attributed to a parallel increase in the concentration of ethanol which is the precursor molecule of this analyte throughout the process (from ca. 9.5–10.5 g L^−1^ ethanol in base and stabilized wines to 11.5–12 g L^−1^ ethanol in sparkling wines). Hence, the increase in the ethanol concentrations could lead to higher ethanolamine amounts. This behavior was specific to this compound while the rest of the amines decreased in concentration from base to sparkling wines (see Figure 1b–d). Although these molecules are considered relatively stable in the wine matrix, long periods of aging can produce a gradual decrease in the concentration of biogenic amines. Besides, physical processes such as lees clarification could also contribute to their depletion.

BAs have been traditionally considered descriptors of food quality and high levels are typically associated with food spoilage or production under poor hygienic conditions. For instance, the biogenic amine index (BAI) that accounts for the overall amine concentration, mainly from histamine, tyramine, putrescine, and cadaverine contributions, is a good parameter to express the freshness of foodstuffs. For fresh products, BAI values between 20 and 50 mg kg^−1^ denote low quality while above 50 mg kg^−1^ is a symptom of product deterioration. In the case of fermented products such as wines, these reference values are not so representative since the typical levels are naturally much higher. Even so, the BA levels in wines and sparkling wines are very low, indicating that the overall quality of these products is good. In the 7-month sparkling wines, the BAI was lower than 3 mg L^−1^ for qualities A and B of both Pinot Noir and Xarel·lo, and lower than 6.4 mg L^−1^ for qualities C and D (except for quality C of Xarel·lo which was 13.3 mg L^−1^). In any case, these are excellent values, so we can assume that the potential adverse effects caused by the consumption of these wines will be negligible. A more exhaustive reference to the potentially harmful effects of BAs is given in some interesting reviews [1,3,30]. The dependence of the BA content on the quality of products has been previously described [7,29] and has also been demonstrated here, as shown in representative examples of Figure 2. Even though the procedures were performed under hygienic and clean conditions for all qualities [3], the highest quality products present lower amine concentrations and vice versa. In part, this finding is due to differences in the quality of the vineyard and the grapes produced, as well as the phytochemical treatments, productivity per hectare, state of fruit maturation, harvesting and transport procedures, and pressing yield.

Beyond the direct formation of BAs, some technological processes can result in another outstanding source of BAs. The mechanized harvesting used in B, C, and D qualities and the high pressing yield and performance obtained for C and D qualities may concurrently lead to wines with high concentrations of malic acid, which provides an unpleasant sour flavor. This circumstance is solved via malolactic fermentation (MLF) to transform malic into lactic acid, with a significant reduction in acidity. Meanwhile, lactic bacteria metabolize the malic acid substrate, together with other organic components, thus resulting in a noticeable rise in the concentration of BAs, especially those more harmful such as histamine. In the samples under study, wines of C and D quality (and to a lower extent some of the B quality) have been subjected to MLF. This multifactorial combination may explain the variation in BA contents as a function of quality, as depicted in the boxplots of Figure 2.

## 4. Materials and Methods

### 4.1. Chemicals

Dansyl-Cl (98%, Acros Organics, Geel, Belgium), acetone (LichroSolv, Merck, Darmstadt, Germany), sodium hydroxide (analytical grade, Merck, Darmstadt, Germany), sodium carbonate (analytical grade, Merck, Darmstadt, Germany), acetonitrile (UHPLC Supergradient grade, AppliChem, Castellar del Vallès, Spain), formic acid (≥95%, ACS, Sigma-Aldrich, Burlington, MA, USA), and purified Milli-Q water (Millipore Corporation, Bedford, MA, USA) were used to prepare reagent, buffer and carrier solutions. Biogenic amines were as follows (unless otherwise indicated, the purity of these compounds is greater than 99%): histamine dihydrochloride, octopamine hydrochloride, tryptamine hydrochloride (≥97%), 2-phenylethylamine hydrochloride, spermidine trihydrochloride, spermine tetrahydrochloride, tyramine hydrochloride (≥97%). Biogenic amines were supplied by Fluka (Buchs, Switzerland). Putrescine dihydrochloride, cadaverine dihydrochloride (98%), and agmatine sulfate (98%) were sourced from Alfa Aesar (Kandel, Germany). 2-aminoethanol hydrochloride (>98%), ethylamine hydrochloride (>98%), and hexylamine hydrochloride were supplied by Tokyo Chemical Industry (TCI, Tokyo, Japan). Stock solutions were stored at −20 °C until use.

Individual stock standard solutions of each BA were prepared in Milli-Q water at a concentration of 1000 mg L^−1^ using the following products. Intermediate aqueous standards (100, 50, and 20 mg L^−1^) and calibration standards (1 to 20 mg L^−1^) were prepared by proper dilution and kept at 2 °C until use. The reagent solution consisted of 50 mg of dansyl-Cl dissolved in 40 mL of acetone. The buffer solution to maintain a proper reaction pH was composed of 1.4 mM NaOH and 0.1 mM Na_2_CO_3_. The carrier solution was water/acetonitrile (1:1, *v*:*v*).

### 4.2. Samples and Sample Treatment

Monovarietal musts, base wines, stabilized wines, and sparkling wines (3- and 7-month-aged) of Xarel·lo (white grape) and Pinot Noir (red grape) varieties were obtained from Raventós Codorníu group (Sant Sadurní d’Anoia, Spain). For each combination, four different qualities were available, here referred to as A, B, C, and D (A is the highest quality, and D is the lowest). The quality standards were defined by the enologists in the field according to agricultural and oenological criteria, including features such as manual or mechanical grape collection, organic or conventional (fertilizers and pesticides) agriculture, productivity per hectare, etc. (additional detail for rating the quality are available online at https://www.mdpi.com/article/10.3390/beverages8010003/s1 (accessed on 6 December 2022) [31]

Samples were filtered through nylon membranes (0.45 µm pore size) and derivatized as described elsewhere. Briefly, 250 µL of the sample, reagent, and buffer solutions were mixed in chromatographic vials. The reaction was developed for 15 min at room temperature and the resulting solution was ready to be analyzed by FIA-MS. Standard solutions and quality controls were derivatized in the same way.

### 4.3. FIA-MS Method

The water: ACN (1:1, *v*/*v*) carrier solution was pumped through the system using an Agilent 1100 binary pump (G1312A, Agilent Technologies, Palo Alto, CA, USA) at a flow rate of 0.4 mL min^−1^. Samples and standards were injected with an Agilent autosampler (G1379A); the injection volume was 20 μL. Analytes were detected by multiple reaction monitoring (MRM) using a hybrid triple quadrupole/linear ion trap mass spectrometer (QTRAP 4000, from Applied Biosystems, AB Sciex, Framingham, MA, USA). Electrospray (ESI) was the ionization source, working in positive mode. Nitrogen was used as a nebulizer and auxiliary gas as well as the collision gas. The source conditions are as follows: Source voltage, 4500 V; source temperature, 500 °C; gas 1 pressure (heating gas at the source), 50 psi; auxiliary gas pressure (drying gas), 50 psi; capillary temperature, 350 °C; curtain gas pressure, 10 psi. Transitions selected and other MS conditions have been detailed in Table S3 (Supplementary Materials). Analyst 6.2 software (AB Sciex) was used to control the instrument and quantify the analytes.

BA standards for calibration were injected at the beginning and end of the FIA-MS sequence. Samples were subsequently injected randomly. Three independent replicates of each sample were analyzed.

## 5. Conclusions

Fast and accurate analytical methods to determine biogenic amines in wines are increasingly in demand to more feasibly monitor the vinification process as well as for assessing toxicological issues. Here, we propose biogenic amine profiling by the FIA-MS method to provide efficient descriptors of wine characteristics and quality issues. In general, overall biogenic amine concentrations are lower than 10 mg L^−1^, so they do not present any potential risk for consumers. Furthermore, levels of amines with unpleasant organoleptic implications, such as putrescine and cadaverine, are low as well, meaning that no flavor defects due to compounds are expected, at least from the point of view of the biogenic amines. Both sample type and sample quality are remarkable factors affecting the levels of amines in the samples. First, there is a rise from musts to wines due to the fermentation processes. Subsequently, amine concentrations generally decay during the aging stages. Regarding quality, it was evident that the best qualities contained significantly lower amine levels. Since concentration differences are not so noticeable in musts, this rise is mainly due to malolactic fermentation that has been applied to wines of lower quality.

## Data Availability

Data is given in the manuscript and Supplementary Materials.

## Supplementary Materials

The following supporting information can be downloaded at: https://www.mdpi.com/article/10.3390/molecules27248690/s1, Figure S1. Structures of the biogenic amines determined in this study. Table S1. Analytical parameters of the FIA-MS/MS method. Table S2. Concentrations and standard deviations (n = 3) of the analyzed biogenic amines in the set of samples determined by FIA-MS. Sample nomenclature: must (M), base wine (BW), stabilized wine (SW), sparkling wine (3 months aged, 3M), sparkling wine (7 months aged, 7M), A-type Pinot Noir (PA), B-type Pinot Noir (PB), C-type Pinot Noir (PC), D-type Pinot Noir (PD), A-type Xarel·lo (XA), B-type Xarel·lo (XB), C-type Xarel·lo (XC), and D-type Xarel·lo (XD). Table S3. Transitions for each analyte and the optimal values of declustering potential (DP), collision energy (CE), cell exit potential (CXP), and retention time of each analyte.

## Sample Availability

Samples are available from the authors.
