# Supplementary material for: Bioactive Amines in Wines. The Assessment of Quality Descriptors by Flow Injection Analysis with Tandem Mass Spectrometry
Source: Molecules. 2022 Dec 8;27(24):8690. doi: 10.3390/molecules27248690 (PMC9783241; doi:10.3390/molecules27248690)
Supplement: Supplementary file 1 [file molecules-27-08690-s001.zip › molecules-2062149-supplementary.pdf]

Supplementary material

Bioactive Amines in Wines. Assessment of Quality Descriptors by Flow Injection Analysis with Tandem Mass Spectrometry

Aina Mir-Cerdà[1,2], Javier Saurina[1,2,*], and Sònia Sentellas[1,2,3]

[1] Department of Chemical Engineering and Analytical Chemistry, Universitat de Barcelona. Martí i Franquès 1-11, E08028, Barcelona, Spain.

[2] Research Institute in Food Nutrition and Food Safety, Universitat de Barcelona, Recinte Torribera, Av. Prat de la Riba 171, Edifici de Recerca (Gaudí), Santa Coloma de Gramenet, E08921 Barcelona, Spain.

[3] Serra Húnter Fellow Programme, Generalitat de Catalunya, Via Laietana 2, E-08003 Barcelona, Spain.

* Correspondence: J.S. xavi.saurina@ub.edu

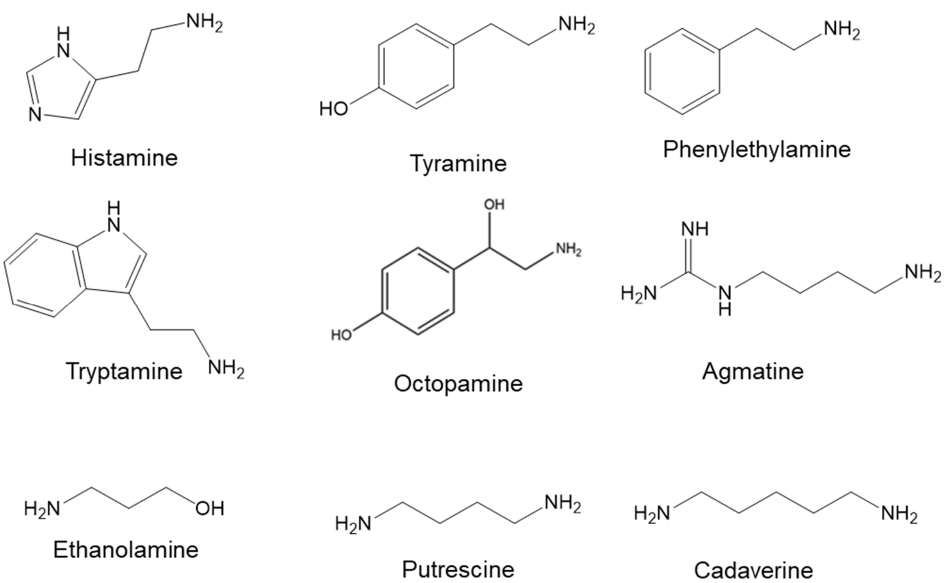

Figure S1. Structures of the biogenic amines determined in this study

Table S1. Analytical parameters of the proposed FIA-MS/MS method.

**Biogenic amine**	**Linear range ($mg\ L^{-1}$)**	**Sensitivity (counts min L mg^{-1})**	**Correlation coefficient**	**LOD ($mg\ L^{-1}$)**	**RSD (%)**
Agmatine	up to 5	48600	0.997	0.20	9.8
Cadaverine	up to 10	19980	0.990	0.19	6.8
Ethanolamine	up to 10	38780	0.980	0.71	7.0
Histamine	up to 10	305000	0.985	0.88	6.8
Octopamine	up to 10	58000	0.990	0.28	6.6
Phenylethylamine	up to 10	63400	0.995	0.12	6.7
Putrescine	up to 25	2342	0.990	0.19	8.1
Tryptamine	up to 10	3274	0.985	0.43	6.8
Tyramine	up to 25	9900	0.980	0.74	5.8

Table S2. Concentrations and standard deviations (n=3) of the analyzed biogenic amines in the set of samples determined by FIA-MS. Sample nomenclature: must (M), base wine (BW), stabilized wine (SW), sparkling wine (3 months aged, 3M), sparkling wine (7 months aged, 7M), A-type Pinot Noir (PA), B-type Pinot Noir (PB), C-type Pinot Noir (PC), D-type Pinot Noir (PD), A-type Xarel·lo (XA), B-type Xarel·lo (XB), C-type Xarel·lo (XC), and D-type Xarel·lo (XD).

**Sample type**	**Ethanolamine**		**Agmatine**		**Tryptamine**		**Phenylethylamine**		**Putrescine**		**Cadaverine**		**Histamine**		**Octopamine**		**Tyramine**	
	Conc. (mg L^{-1})	s	Conc. (mg L^{-1})	s	Conc. (mg L^{-1})	s	Conc. (mg L^{-1})	s	Conc. (mg L^{-1})	s	Conc. (mg L^{-1})	s	Conc. (mg L^{-1})	s	Conc. (mg L^{-1})	s	Conc. (mg L^{-1})	s
**MPA**	0.13	0.07	0.11	0.06	0.14	0.01	0.03	0.01	0.06	0.00	0.06	0.03	0.07	0.02	0.10	0.06	0.29	0.25
**BWPA**	2.39	0.14	0.90	0.19	0.49	0.07	0.49	0.16	3.54	1.44	0.41	0.07	0.77	0.20	1.68	0.34	2.74	0.31
**SWPA**	2.05	0.12	0.98	0.07	0.45	0.10	0.38	0.12	3.67	0.18	0.34	0.07	0.54	0.11	1.44	0.39	2.17	0.65
**3MPA**	2.74	0.36	0.54	0.20	0.44	0.02	0.13	0.01	0.79	0.30	0.15	0.06	0.39	0.14	0.71	0.17	0.78	0.18
**7MPA**	2.62	0.47	0.39	0.05	0.44	0.02	0.09	0.02	0.14	0.02	0.12	0.01	0.14	0.06	0.49	0.04	0.64	0.09
**MXA**	0.78	0.15	0.53	0.15	0.18	0.03	0.04	0.01	0.14	0.04	0.23	0.02	0.23	0.08	0.54	0.12	0.84	0.41
**BWXA**	3.38	0.47	1.00	0.03	0.52	0.14	0.49	0.22	2.03	0.48	0.43	0.01	0.32	0.04	1.62	0.29	3.79	1.04
**SWXA**	2.89	1.04	0.91	0.19	0.71	0.09	0.45	0.09	1.62	0.44	0.31	0.03	0.52	0.32	1.33	0.26	4.92	2.51
**3MXA**	4.38	1.89	0.58	0.03	0.55	0.11	0.10	0.02	0.44	0.10	0.16	0.03	0.27	0.02	0.77	0.08	0.82	0.13
**7MXA**	3.34	0.26	0.47	0.01	0.48	0.10	0.09	0.01	0.21	0.01	0.12	0.02	0.18	0.05	0.76	0.09	0.64	0.15
**MPB**	0.43	0.29	0.44	0.15	0.14	0.01	0.04	0.01	0.06	0.03	0.09	0.07	0.19	0.09	0.31	0.22	0.96	0.63
**BWPB**	4.07	0.14	1.39	0.30	0.54	0.05	0.63	0.16	2.37	0.41	0.80	0.21	0.82	0.18	2.54	0.54	11.84	2.83
**SWPB**	4.22	0.33	1.06	0.20	0.29	0.13	0.47	0.08	2.59	0.43	0.44	0.10	1.24	0.32	1.58	0.15	8.92	2.14
**3MPB**	6.17	0.13	0.65	0.08	0.38	0.05	0.16	0.02	0.77	0.13	0.19	0.01	0.56	0.05	0.89	0.12	1.04	0.01
**7MPB**	6.39	0.70	0.39	0.14	0.38	0.05	0.08	0.04	0.23	0.08	0.13	0.06	0.30	0.10	0.79	0.24	0.59	0.23
**MXB**	0.38	0.11	0.20	0.02	0.05	0.08	0.02	0.00	0.35	0.07	0.07	0.01	0.07	0.05	0.19	0.09	5.08	2.61
**BWXB**	4.47	0.42	0.85	0.16	0.56	0.05	0.53	0.17	2.37	0.10	0.39	0.08	0.36	0.05	1.44	0.14	2.90	0.48
**SWXB**	5.02	0.60	1.02	0.23	0.35	0.07	1.15	0.11	23.71	3.51	2.09	0.93	13.41	4.70	2.29	0.85	17.01	6.48
**3MXB**	5.46	1.03	0.42	0.11	0.29	0.03	0.09	0.00	0.99	0.37	0.16	0.08	0.44	0.19	0.40	0.12	0.44	0.05
**7MXB**	6.14	2.14	0.28	0.01	0.34	0.14	0.08	0.01	0.17	0.04	0.07	0.01	0.25	0.12	0.38	0.03	0.46	0.06

**MPC**	1.06	0.03	0.48	0.18	0.05	0.08	0.08	0.01	0.24	0.13	0.18	0.07	0.26	0.16	0.58	0.19	1.21	0.56
**BWPC**	4.44	0.32	1.80	0.15	0.77	0.19	1.01	0.14	23.75	6.51	0.85	0.08	14.69	1.78	5.15	1.01	23.54	2.08
**SWPC**	4,19	0,38	1,01	0,10	0.32	0.04	0,61	0,11	9,81	3,20	0,60	0,10	6,70	0,90	2,01	0,44	11,42	2,54
**3MPC**	5,36	0,79	0,38	0,08	0.24	0.01	0,25	0,02	3,08	0,16	0,13	0,01	1,51	0,13	0,45	0,09	2,48	0,42
**7MPC**	6,07	0,57	0,28	0,06	0.24	0.01	0,22	0,05	0,95	0,99	0,12	0,06	0,63	0,58	0,46	0,05	1,97	0,55
**MXC**	2,04	0,71	0,86	0,30	0.25	0.02	0,07	0,02	0,64	0,29	0,31	0,07	0,21	0,09	0,93	0,31	1,32	0,19
**BWXC**	4,74	0,61	0,92	0,49	0.45	0.04	0,76	0,37	7,72	5,22	1,16	0,61	4,98	2,91	2,09	1,28	14,69	4,33
**SWXC**	4,92	0,21	1,14	0,33	0.33	0.06	0,73	0,19	12,97	3,54	0,94	0,41	9,79	2,19	1,68	0,19	14,27	0,59
**3MXC**	6,57	0,80	0,46	0,02	0.31	0.02	0,28	0,01	2,57	0,35	0,19	0,03	1,41	0,38	0,47	0,01	1,43	0,29
**7MXC**	6,72	0,83	0,30	0,01	0.32	0.04	0,21	0,02	1,25	0,64	0,10	0,04	0,48	0,09	0,45	0,05	10,23	6,46
**MPD**	0,80	0,26	0,53	0,10	0.15	0.01	0,07	0,03	0,20	0,07	0,15	0,01	0,29	0,16	0,58	0,07	1,19	0,38
**BWPD**	4,61	0,46	0,89	0,08	0.34	0.04	0,93	0,13	15,93	4,35	0,78	0,24	9,55	3,00	3,20	1,63	22,89	5,67
**SWPD**	4,61	0,28	0,96	0,04	0.28	0.03	1,14	0,07	22,91	2,90	1,04	0,18	16,66	5,58	3,97	0,83	31,89	2,85
**3MPD**	5,82	0,29	0,41	0,17	0.25	0.03	0,34	0,04	3,82	1,70	0,14	0,06	2,02	0,93	0,33	0,13	0,99	0,74
**7MPD**	6,28	0,69	0,38	0,03	0.25	0.03	0,28	0,04	0,49	0,08	0,08	0,01	0,44	0,14	0,43	0,03	2,84	2,36
**MXD**	0,44	0,06	0,33	0,15	0.09	0.08	0,04	0,00	1,47	0,58	0,14	0,08	0,06	0,06	0,35	0,19	0,81	0,09
**BWXD**	4,80	0,70	0,74	0,34	0.54	0.03	0,68	0,29	6,03	1,31	0,15	0,09	4,34	3,29	1,11	0,71	11,42	1,19
**SWXD**	5,23	0,75	0,95	0,22	0.26	0.03	0,67	0,15	7,97	2,11	1,20	0,46	5,32	2,22	2,94	0,77	10,93	1,26
**3MXD**	7,03	0,46	0,39	0,05	0.30	0.02	0,25	0,00	2,09	0,82	0,18	0,06	1,42	0,43	0,44	0,08	1,19	0,61
**7MXD**	7,37	1,23	0,38	0,02	0.32	0.16	0,32	0,01	0,74	0,29	0,10	0,05	0,59	0,12	0,52	0,07	3,42	1,17

Table S3. Transitions for each analyte and the optimal values of declustering potential (DP), collision energy (CE), cell exit potential (CXP), and retention time of each analyte.

Biogenic amine	Q1 mass (Da)	Q3 mass (Da)	DP (V)	CE (V)	CXP (V)
Agmatine	597.0	170.0	50	80	8
Tyramine	604.0	234.0	90	40	8
Phenylethylamine	355.0	170.0	90	30	10
Cadaverine	569.0	234.0	110	50	8
Putrescine	555.0	234.0	120	60	12
Histamine	578.0	234.0	80	40	12
Tryptamine	394.0	234.0	60	40	10
Spermidine	845.0	360.0	40	80	12
Spermine	1135.6	360.0	90	50	10
Octopamine	620.0	170.0	66	59	12
Ethanolamine	295.0	170.0	120	60	12
